# Changes in Soil Nutrients and Bacterial Communities in Perennial Grass Mixtures in Alpine Ecological Zones After 20 Years of Establishment

**DOI:** 10.3390/plants15050754

**Published:** 2026-02-28

**Authors:** Shancun Bao, Zongcheng Cai, Fayi Li, Hairong Zhang, Shouquan Fu, Liangyu Lv, Qingqing Liu, Jianjun Shi

**Affiliations:** 1 Academy of Animal Science and Veterinary, Qinghai University, Xining 810016, China; yb240909000115@qhu.edu.cn (S.B.);; 2 Key Laboratory of Alpine Grassland Ecology in the Three-River-Source Region, Ministry of Education, Xining 810016, China; 3 Qinghai Provincial Key Laboratory of Adaptive Management of Alpine Grasslands, Ministry of Education, Xining 810016, China

**Keywords:** alpine region, artificial grassland, grass species diversity, soil organic matter, Bacterial community structure

## Abstract

Monoculture and mixed sowing are common practices for restoring degraded alpine meadow grasslands. To investigate the effects of different sowing patterns on soil bacterial community characteristics in alpine artificial grasslands, this study examined a 20-year-old established artificial grassland, systematically analyzing plant community attributes, soil physicochemical properties, and the diversity and functional structure of soil bacterial communities under various monoculture and mixed-sowing treatments. The results showed that: (1) Mixed-sowing treatments significantly improved soil physicochemical properties and plant community characteristics. The P4 (*Elymus nutans* + *Poa pratensis* + *Festuca sinensis* + *Poa crymophila*) mixed-sowing treatment notably enhanced vegetation performance and soil conditions. Compared with the monoculture P1 (*Elymus nutans*) treatment, aboveground biomass (AGB) and soil organic matter (SOM) content increased by 57.23% and 68.25%, respectively, indicating that perennial grass mixtures improve soil water and nutrient retention, thereby promoting plant growth. (2) Microbiome analysis revealed that mixed sowing significantly optimized the structure of rhizosphere bacterial communities. Operational Taxonomic Units (OTUs), which represent sequence-based taxonomic units and their abundance information, were most abundant in the P4 mixed-sowing treatment, reaching a total of 5685 OTUs. In terms of bacterial diversity indices, the OTU richness, Ace index, and Chao1 index in the P4 mixed-sowing treatment were 26.12%, 25.81%, and 24.34% higher, respectively, than those in the monoculture P1 treatment, with all differences being statistically significant (*p* < 0.05). (3) Mantel test and redundancy analysis (RDA) revealed that soil electrical conductivity (SEC) and pH were negatively correlated with bacterial diversity indices, while soil organic matter (SOM) was identified as the key environmental driver shaping bacterial community assembly. In summary, appropriate grass mixtures effectively enhance “plant–soil–microbe” interactions, leading to improved soil fertility and optimized bacterial communities, representing a viable strategy for long-term ecological restoration and sustainability of alpine artificial grassland ecosystems. The P4 treatment—comprising a four-species mixture of *Elymus nutans*, *Poa pratensis*, *Poa crymophila*, and *Festuca sinensis*—achieved the best overall performance.

## 1. Introduction

Alpine grassland ecosystems play an irreplaceable role in global carbon cycling, water conservation, and biodiversity maintenance [1]. However, excessive grazing, climate warming, and freeze–thaw erosion have led to degradation of more than 60% of natural grasslands, severely threatening regional ecological security and the livelihoods of herders [2]. Therefore, restoring degraded grasslands has become an urgent issue, and selecting appropriate ecological restoration measures is critical for effective rehabilitation of degraded ecosystems. Restoration measures for degraded alpine grasslands primarily include plowing, reseeding, fertilization, mowing, artificial grassland establishment, fencing with grazing exclusion, and regulated grazing. Among these, artificial grassland establishment is particularly critical for severely degraded grasslands. It not only effectively promotes ecological recovery of degraded ecosystems but is also considered one of the most effective approaches for rehabilitating “black-soil patches” [3].

Currently, widely applied artificial grassland establishment techniques can significantly improve plant community cover, plant height, biomass, and species diversity in the short term [4]. Relevant studies have shown that artificial grasslands established by plowing and seeding perennial grasses can achieve vegetation cover exceeding 80% within two years [5]. In artificial grassland establishment, monoculture (single-species sowing) and mixture sowing (polyculture) are the two most commonly used seeding approaches, both of which effectively enhance vegetation cover and productivity [6]. Monoculture often exhibits high productivity, whereas in mixed sowing systems, different grass species occupy distinct ecological niches. Among these, mixtures of perennial grasses have received increasing attention due to their interspecific complementarity and strong stress tolerance [7]. By scientifically selecting complementary grass species for mixture sowing, soil structure can be significantly improved, surface coverage enhanced, water and soil loss regulated, and both grassland productivity and ecological stability increased. Moreover, such mixtures can effectively prevent secondary degradation [8].

However, beyond plant community characteristics, soil physicochemical properties and soil microbial communities also play crucial roles in the ecological restoration outcomes of artificial grasslands and in the process of secondary degradation [9]. Soil microorganisms, as key decomposers in grassland ecosystems, are extensively involved in the transformation and biogeochemical cycling of carbon, nitrogen, phosphorus, and other elements. They serve as a vital link between aboveground vegetation and belowground processes, providing diverse ecological functions [10]. The establishment of artificial grasslands can significantly influence soil physical structure and the composition and function of microbial communities by increasing belowground biomass and altering root morphology and distribution. Furthermore, through regulating the types and quantities of root exudates, artificial grasslands can affect the potential functions of the soil microbiome in nutrient cycling and pathogen suppression [11]. Previous studies have demonstrated that, compared to monoculture artificial grasslands, mixed-sowing grasslands exhibit higher bacterial diversity and more complex microbial interaction network structures. Additionally, soil total nitrogen, total phosphorus, organic carbon, as well as microbial biomass carbon, nitrogen, and phosphorus, are significantly increased in mixed sowing systems. The structural characteristics, functional potential, and ecological network stability of soil microbial communities have thus been widely adopted as key indicators for evaluating the effectiveness of different vegetation restoration strategies in rehabilitating degraded alpine grasslands [12]. However, systematic studies on the effects of monoculture versus mixed sowing patterns on soil bacterial community structure and functional characteristics are still lacking, and the underlying mechanisms remain unclear. Therefore, this study focuses on a 20-year-old artificial grassland established with monocultured perennial grasses and mixed-sown perennial grasses at different composition ratios. By comparing soil physicochemical properties and the composition, structure, and functional characteristics of soil bacterial communities under different planting patterns, this research aims to reveal the long-term dynamics of bacterial community succession and their responses to soil physicochemical factors following artificial restoration. The study specifically addresses two key scientific questions: (1) The characteristics of soil bacterial community composition and co-occurrence network structure in perennial grass mixtures with different species ratios over a 20-year period of natural succession; (2) The interactive relationships between key bacterial taxa and soil environmental variables. The findings will provide important theoretical insights and technical support for the scientific restoration, stability maintenance, and sustainable management of degraded alpine grassland ecosystems.

## 2. Materials and Methods

### 2.1. Study Area Description

**Figure 1:**
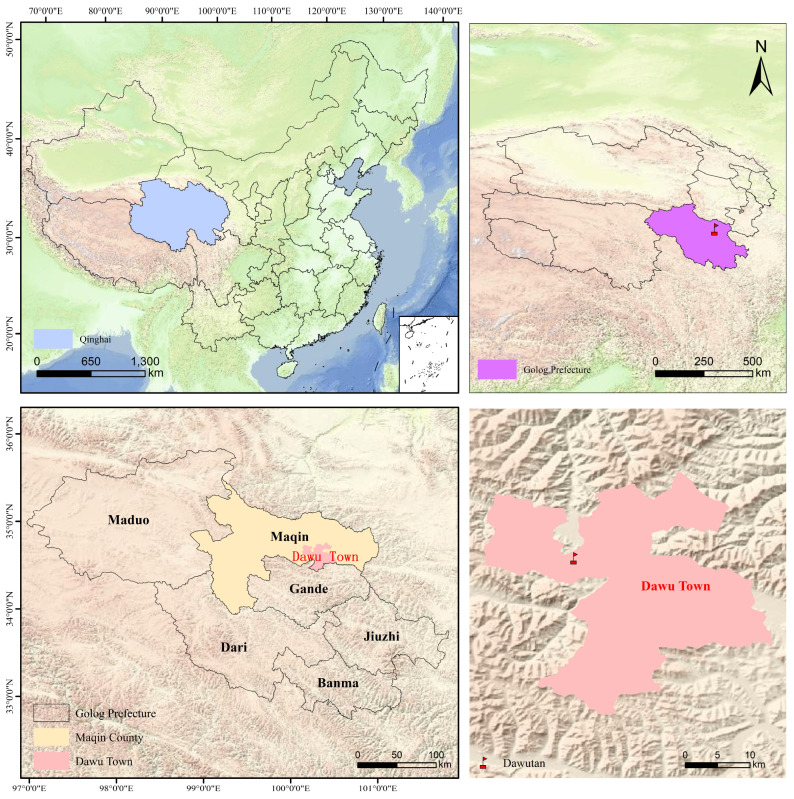
Geographical location of the experimental area.

The study site is located in Maqin County, Guoluo Tibetan Autonomous Prefectu (Figure 1), Qinghai Province, China (34°46′41″ N, 100°21′26″ E), with an average elevation of 3760 m and a relatively flat topography. The area is characterized by a typical plateau continental climate, with small annual temperature variation but large diurnal fluctuations and intense solar radiation. Long-term meteorological observations indicate a mean annual air temperature of −3.9 °C, with monthly temperatures ranging from −14.8 °C in January to 9.8 °C in July. Precipitation is highly seasonal, with approximately 60% occurring during the summer months, while winter precipitation accounts for only 1–2% of the annual total. The mean annual precipitation ranges from 500 to 600 mm, whereas annual evaporation reaches up to 1500 mm. The growing season for grasses lasts approximately 156 days, and there is no absolute frost-free period. The dominant soil type is alpine meadow soil, and the vegetation is primarily composed of alpine meadow communities [13].

### 2.2. Experimental Design and Methods

The experiment was conducted in a long-term observational artificial grassland established in 2006 within a uniform field site. Prior to establishment, the soil conditions, vegetation type, and degraded “Heitutan” (degraded alpine meadow) characteristics were consistent across the experimental area. Standard establishment practices included plowing, harrowing, broadcasting of seeds, and fencing to exclude grazing for natural regeneration. Six perennial grass species were selected for the experiment, all of which are native species that have been domesticated locally and are widely promoted and cultivated in the region. These species were used to establish both monoculture and mixed-sowing plots to evaluate the long-term effects of different planting patterns on soil properties and microbial communities. Among the six perennial grass species used, *Elymus nutans*, *Poa pratensis* cv. Qinghai, *Poa crymophila* cv. Qinghai, *Festuca sinensis*, *Puccinellia distans*, and *Festuca kryloviana* cv. Huanhu were all propagated from seeding materials derived from local introduction and domestication trials. All species were sown following standard guidelines for artificial grassland establishment in Qinghai Province. In each treatment, the seeding rate for each grass species was calculated as the monoculture seeding rate divided by the number of species in the mixture (*n*). The monoculture seeding rate was 3.0 g·m^−2^ for *Elymus nutans*, 0.75 g·m^−2^ for *Poa pratensis*, *Poa crymophila*, *Puccinellia distans*, and *Festuca kryloviana* cv. Huanhu, and 2.25 g·m^−2^ for *Festuca sinensis*. A completely randomized block design was employed in this study, with three blocks. Each block contained six experimental plots (3 m × 4 m), assigned to six sowing treatments (P1, P2, P3, P4, P5, P6) arranged randomly. Each treatment was applied once per block, with plot positions randomized within blocks. Buffer zones 1.0 m wide were established between adjacent plots, and 2.0 m-wide isolation strips were set between blocks to minimize edge effects and treatment interference. Specific seeding rates and mixture configurations are detailed in Table 1.

### 2.3. Plant Community Survey and Soil Sample Collection

In August 2024 and the corresponding period in 2025, community height, coverage, density, and aboveground biomass were surveyed. Within each experimental plot, five 0.5 m × 0.5 m quadrats were randomly established. Vegetation height was measured using a tape measure (precision: 1 mm), with 15 individual plants measured for each grass species, and the mean value recorded as the vegetation height. Species number and plant counts within each quadrat were recorded by direct observation and counting to calculate vegetation density and community diversity indices. Community coverage was determined using the point-intercept method. Aboveground vegetation within each quadrat was harvested at ground level, weighed to obtain fresh weight, and then samples were oven-killed at 105 °C for 30 min, followed by drying at 65 °C for 48 h to constant weight for determination of dry biomass.

Soil sampling was conducted simultaneously. Soil samples from the 0–10 cm layer were collected using a 5 cm diameter soil auger following a five-point composite sampling method. In each plot, the five subsamples were mixed thoroughly to form one composite soil sample, which was then divided equally into three subsamples: one subsample was air-dried in the dark for analysis of soil physicochemical properties; one fresh subsample was used immediately to measure soil gravimetric moisture content, pH, and electrical conductivity (SEC); and one fresh subsample was stored at −80 °C for subsequent analysis of soil microbial community diversity.

### 2.4. Measurement of Indicators and Methods

#### 2.4.1. Soil Physicochemical Properties Analysis

The soil physicochemical properties, including soil water content (SWC), soil electrical conductivity (SEC), pH, soil organic matter (SOM), total nitrogen (TN), total phosphorus (TP), alkali-hydrolyzable nitrogen (AN), and available phosphorus (AP), were determined following the standard procedures outlined in Soil Agrochemical Analysis (3rd Edition) [14].

#### 2.4.2. Soil Bacterial DNA Extraction, PCR Amplification, and Sequencing

Soil samples were sent to Majorbio Bio-Pharm Technology Co., Ltd. (Shanghai, China) for sequencing. The V3–V4 region of the bacterial 16S rDNA gene was amplified using primers 338F (5′-ACTCCTACGGGAGGCAGCA-3′) and 806R (5′-GGACTACHVGGGTWTCTAAT-3′). Paired-end sequencing (PE300 mode) was conducted on the Illumina MiSeq 6000 platform (Illumina, San Diego, CA, USA) by Majorbio Bio-Pharm Technology Co., Ltd. (Shanghai, China). The sequencing workflow included the following steps: extraction of total soil DNA, quality assessment of genomic DNA, PCR amplification, agarose gel electrophoresis of PCR products, purification of PCR products, construction of MiSeq libraries, library quality inspection, and sequencing on the Illumina MiSeq platform. Raw sequencing data were subsequently processed and analyzed using the QIIME2 (QIIME 2 release: 2024.5) platform. To investigate taxonomic composition, effective tags from all samples were clustered into operational taxonomic units (OTUs) at a 97% sequence identity threshold, followed by taxonomic annotation using the UNITE database (v8.2) with a confidence threshold of 80%. All downstream analyses were performed on the Majorbio Cloud Platform (www.majorbio.com) to ensure computational reproducibility. Microbial α-diversity was characterized using multiple indices, including OTU richness (the number of observed OTUs per sample), Shannon, Ace, Chao1, Simpson, and Pielou indices. β-diversity of fungal communities was evaluated using principal coordinate analysis (PCoA) based on Bray–Curtis dissimilarity, and statistical significance was assessed via permutational multivariate analysis of variance (PERMANOVA). Linear discriminant analysis effect size (LEfSe) was employed to identify differentially abundant taxa at the phylum and genus levels. Fungal co-occurrence networks were constructed using the “Network Analysis” pipeline on the Majorbio Cloud Platform. To ensure statistical robustness and minimize noise, the top 50 most abundant fungal taxa were selected as nodes. Pairwise correlations were calculated using Spearman’s rank correlation coefficient, and significant edges were defined as those with |r| ≥ 0.5 and *p* < 0.05. Topological properties of the networks—including number of nodes, number of edges, and proportions of positive and negative correlations—were visualized and analyzed to reflect the complexity and stability of the microbial community. Fungal functional guilds were predicted using FUNGuild v1.2 on the Majorbio Cloud Platform (https://cloud.majorbio.com).

### 2.5. Data Analysis and Graphical Visualization

Raw data were organized using Microsoft Excel 2019, and statistical analyses were performed using SPSS 25.0. One-way analysis of variance (ANOVA) was applied to vegetation parameters, soil physicochemical properties, and microbial community data. When significant differences among groups were detected (*p* < 0.05), post hoc multiple comparisons were conducted using Duncan’s test at α = 0.05. Redundancy analysis (RDA) was performed in Canoco 5.0 to examine relationships among plant community characteristics, soil physicochemical properties, and soil bacterial diversity. Pearson correlation-based heatmaps illustrating the relationships between aboveground biomass and soil/microbial parameters were generated using the OmicStudio tool (https://www.omicstudio.cn). All final figures were produced using Origin 2022 (OriginLab, Northampton, MA, USA).

## 3. Results and Analysis

### 3.1. Effects of Monoculture and Mixed Sowing of Grasses on Vegetation Community Characteristics

Mixed sowing of different grass species increased plant height, canopy cover, and aboveground biomass, and altered vegetation community diversity compared to monoculture (Table 2). The Simpson index ranged from 0.82 to 0.88 across treatments, with the P4 treatment being significantly lower than the monoculture P1 treatment (*p* < 0.05). Although no significant differences in Shannon and Pielou indices were observed among the mixed-sowing treatments, both indices were significantly different from those in the monoculture P1 treatment (*p* < 0.05). Species richness was highest in the P3 treatment (2.59), significantly greater than in the P1 treatment (*p* < 0.05). The P4 treatment had the highest plant height (32.53 cm) and aboveground biomass (375.80 g·m^−2^), both significantly exceeding those of the P1 treatment (*p* < 0.05). Canopy cover in the mixed-sowing treatments ranged from 81.13% to 88.33%, significantly higher than that in the P1 monoculture (*p* < 0.05). Plant density was highest in the P5 treatment, followed by the P4 treatment.

### 3.2. Effects of Monoculture and Mixed Sowing of Grasses on Soil Physicochemical Properties in Artificial Grassland

Mixed sowing of different grass species increased soil water content and nutrient contents of carbon, nitrogen, and phosphorus, altered soil pH, and reduced soil electrical conductivity compared to monoculture (Table 3). SWC in the mixed-sowing treatments ranged from 21.30% to 25.44%, which was significantly higher than that in the monoculture P1 treatment (*p* < 0.05), with increases ranging from 17.61% to 40.47%. SEC was lowest in the P4 treatment (375.70 μS·cm^−1^), representing a significant reduction of 31.97% compared to P1 (*p* < 0.05). Although soil pH varied slightly among treatments, no statistically significant differences were observed (*p* > 0.05). The P4 treatment exhibited the highest levels of SOM, TN, and AN, reaching 145.05 g·kg^−1^, 6.92 g·kg^−1^, and 61.50 mg·kg^−1^, respectively—representing significant increases of 68.25%, 73.87%, and 40.22% compared to P1 (*p* < 0.05). For phosphorus-related variables, the P6 treatment showed the highest concentrations of TP and AP, at 2.40 g·kg^−1^ and 43.57 mg·kg^−1^, respectively—significantly higher than P1 by 63.27% and 27.58% (*p* < 0.05).

### 3.3. Effects of Monoculture and Mixed Sowing of Grasses on Soil Bacterial Community Composition in Artificial Grassland

#### 3.3.1. Quality Assessment of 16S Sequencing Data and Variation in OTU Numbers

**Figure 2:**
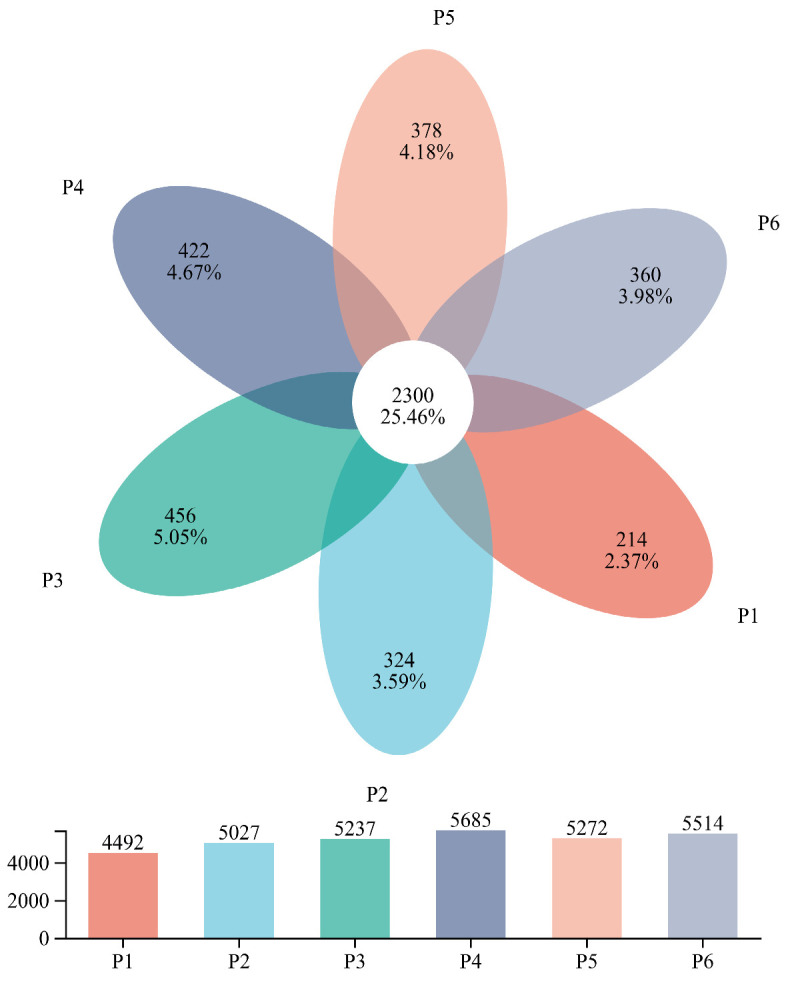
Venn diagram of soil bacterial operational taxonomic units (OTUs) across different treatments.

As shown in the Venn analysis (Figure 2), a total of 9035 soil bacterial OTUs were detected across the six treatments, with 2300 OTUs shared among all treatment groups, indicating a high degree of similarity in bacterial communities across treatments. In contrast, the number of treatment-specific OTUs was relatively low. The number of treatment-specific OTUs in groups P1–P6 was 214, 324, 456, 422, 378, and 360, respectively. In terms of total OTU count, the P4 treatment had the highest number (5685 OTUs), followed by P6 and P5 with 5514 and 5272 OTUs, respectively.

#### 3.3.2. Composition and Relative Abundance of Soil Bacterial Community

**Figure 3:**
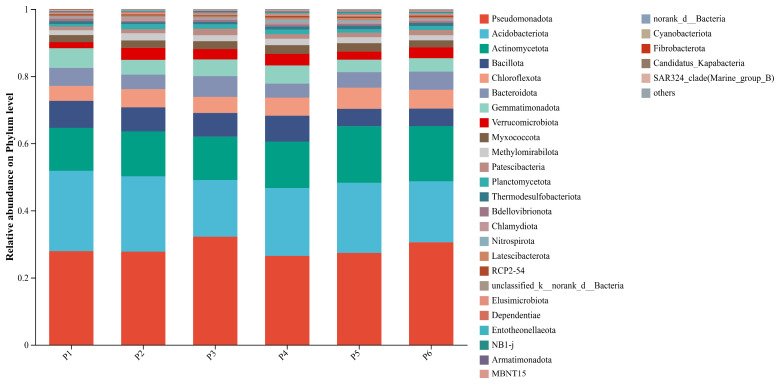
Relative abundance of soil bacterial communities at the phylum level under different treatments.

At the phylum level, the top 31 bacterial taxa with the highest relative abundances across different grass sowing treatments are shown in Figure 3. Dominant bacterial phyla included *Pseudomonadota* (26.46–32.22%), *Acidobacteriota* (16.84–23.93%), *Actinomycetota* (12.77–16.88%), *Bacillota* (5.20–8.03%), *Chloroflexi* (4.63–5.44%), *Bacteroidota* (4.13–6.17%), and *Gemmatimonadota* (3.74–5.82%). These phyla consistently exhibited high relative abundances across all six treatment groups and constituted the core components of the soil bacterial community. Compared to the monoculture treatment (P1), all five mixed-sowing treatments significantly increased the relative abundances of *Actinomycetota* and *Acidobacteriota*. Notably, treatments P3 and P6 enhanced the abundance of *Pseudomonadota*, increasing by 15.44% and 9.35%, respectively, relative to P1.

**Figure 4:**
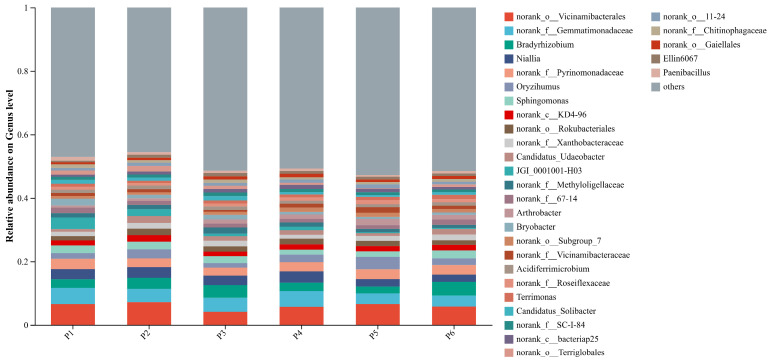
Relative abundance of soil bacterial communities at the genus level under different treatments.

At the genus level, the relative abundances of soil bacterial taxa across the six treatments (Figure 4) were dominated by several prevalent groups, including norank_o__*Vicinamibacterales* (4.12–7.17%), norank_f__*Gemmatimonadaceae* (3.31–5.10%), *Bradyrhizobium* (2.23–4.32%), *Niallia* (2.28–3.55%), norank_f__*Pyrinomonadaceae* (2.54–3.24%), *Oryzihumus* (1.38–3.92%), and *Sphingomonas* (1.55–2.47%). These genera exhibited relatively high abundances and showed minor variation across all treatments, indicating a degree of consistency in the core bacterial genera within the soil microbial community. Compared to the monoculture treatment (P1), all five mixed-sowing treatments reduced the relative abundances of norank_f__*Pyrinomonadaceae* and *Sphingomonas*. Notably, treatments P2 and P5 increased the abundance of norank_o__*Vicinamibacterales*, with increases of 9.47% and 0.76%, respectively, relative to P1.

### 3.4. Analysis of Soil Bacterial Community Diversity

#### 3.4.1. α-Diversity Analysis of Soil Bacterial Communities

**Figure 5:**
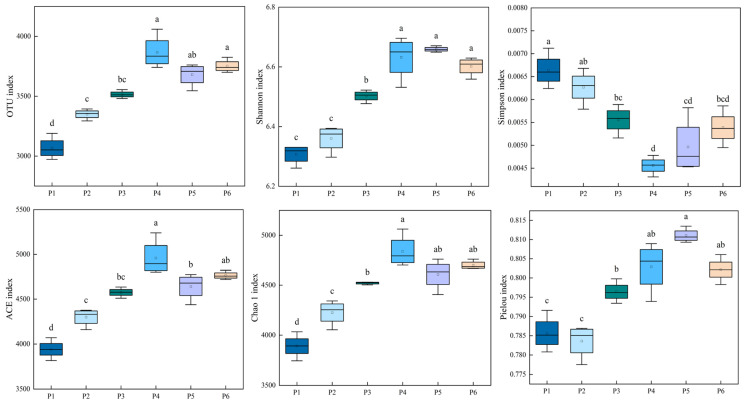
Differences in α-diversity of soil bacterial communities under different treatments. Note: Different lowercase letters in the figure indicate significant differences among treatments at the *p* < 0.05 level.

There were significant differences in the α-diversity indices of soil bacterial communities across the six treatments (Figure 5). The P4 exhibited the highest values for OTU number, Ace index, and Chao1 index, reaching 3867.67, 4957.81, and 4837.77, respectively—representing significant increases of 26.12%, 25.81%, and 24.34% compared to the P1 treatment (*p* < 0.05). The Pielou evenness index was highest in the P5 treatment (0.81), followed by P6 (0.80), which were 4.12% and 2.10% higher than that of P1, respectively. The Simpson index, indicating dominance, decreased in the order: P1 > P2 > P3 > P6 > P5 > P4, with the P4 treatment showing the lowest Simpson index, suggesting the most even bacterial community structure among all treatments.

#### 3.4.2. β-Diversity (PCoA) Analysis of Soil Bacterial Communities

**Figure 6:**
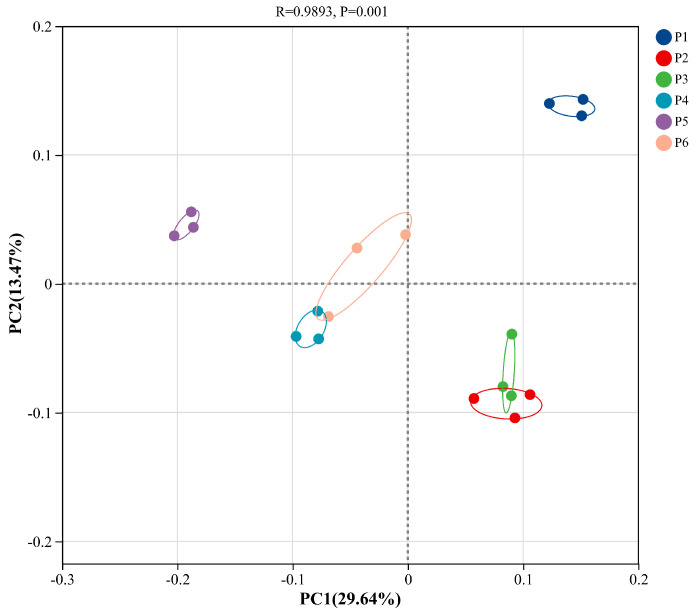
Differences in β-diversity of soil bacterial communities among treatments based on principal coordinate analysis (PCoA).

As shown in Figure 6, PCoA analysis based on Bray–Curtis dissimilarity revealed that the P1 and P2 treatments exhibited the highest clustering and the smallest within-group variation. PC1 explained 29.64% of the variation, and PC2 explained 13.47%, with a cumulative explained variance of 43.11%. Significant differences among groups were detected (*p* = 0.001), and the communities were distinctly clustered into four groups: P1 and P5 each formed a separate cluster, while the P4 and P6 treatments clustered together, and the P2 and P3 treatments formed another cluster.

### 3.5. Changes in Single-Factor Correlation Networks of Soil Bacterial Communities

**Figure 7:**
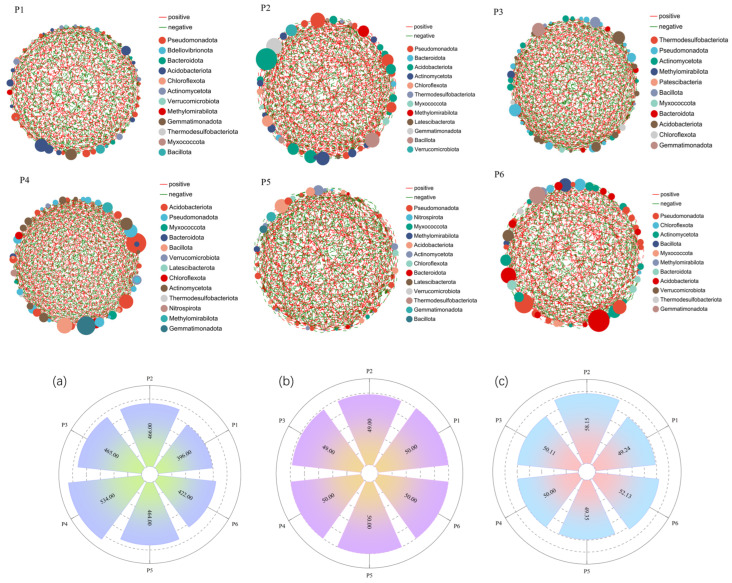
Single-factor correlation networks of soil bacterial communities under different treatments and their topological properties. Note: node color and size indicate species type and importance; line color indicates positive or negative correlation; and red positive, green negative, and the number of lines indicate whether the species are closely related. (**a**) Edge count, (**b**) Node count, (**c**) Positive correlation coefficient.

To investigate the co-occurrence patterns and interactive relationships of soil bacterial communities at the genus level under monoculture and mixed-sowing treatments, single-factor correlation networks were constructed based on genus-level relative abundances (Figure 7). The results revealed significant differences in network topological structures among the treatments. In the bacterial ecological network, the dominant phyla serving as key connector nodes in the P1 monoculture treatment included *Pseudomonadota*, *Bdellovibrionota*, *Bacteroidota*, and *Acidobacteriota*. In contrast, the five mixed-sowing treatments shared a broader set of keystone taxa, with key nodes primarily belonging to *Pseudomonadota*, *Bacteroidota*, *Chloroflexota*, *Acidobacteriota*, *Actinomycetota*, *Bacillota*, *Myxococcota*, and *Methylomirabilota*, indicating a shift in network centrality toward a more diverse assemblage of bacterial groups under mixed planting regimes.

The number of edges in the networks ranged from 396 to 534 across treatments (Figure 7a), showing significant variation, while the number of nodes (representing bacterial genera) was relatively stable, ranging from 49 to 50 (Figure 7b). This suggests that changes in network complexity were primarily driven by shifts in inter-taxa interactions rather than differences in taxon richness. Notably, the proportion of positive correlations varied significantly among treatments (Figure 7c). The P2 treatment exhibited the highest positive correlation ratio (58.15%), followed by P6 (52.13%). In contrast, P1 and P5 showed lower positive correlation ratios, at 49.24% and 49.35%, respectively, both below 50%. A lower proportion of positive interactions may indicate increased competition or reduced community stability under these treatments.

### 3.6. Functional Prediction of Soil Bacterial Communities

**Figure 8:**
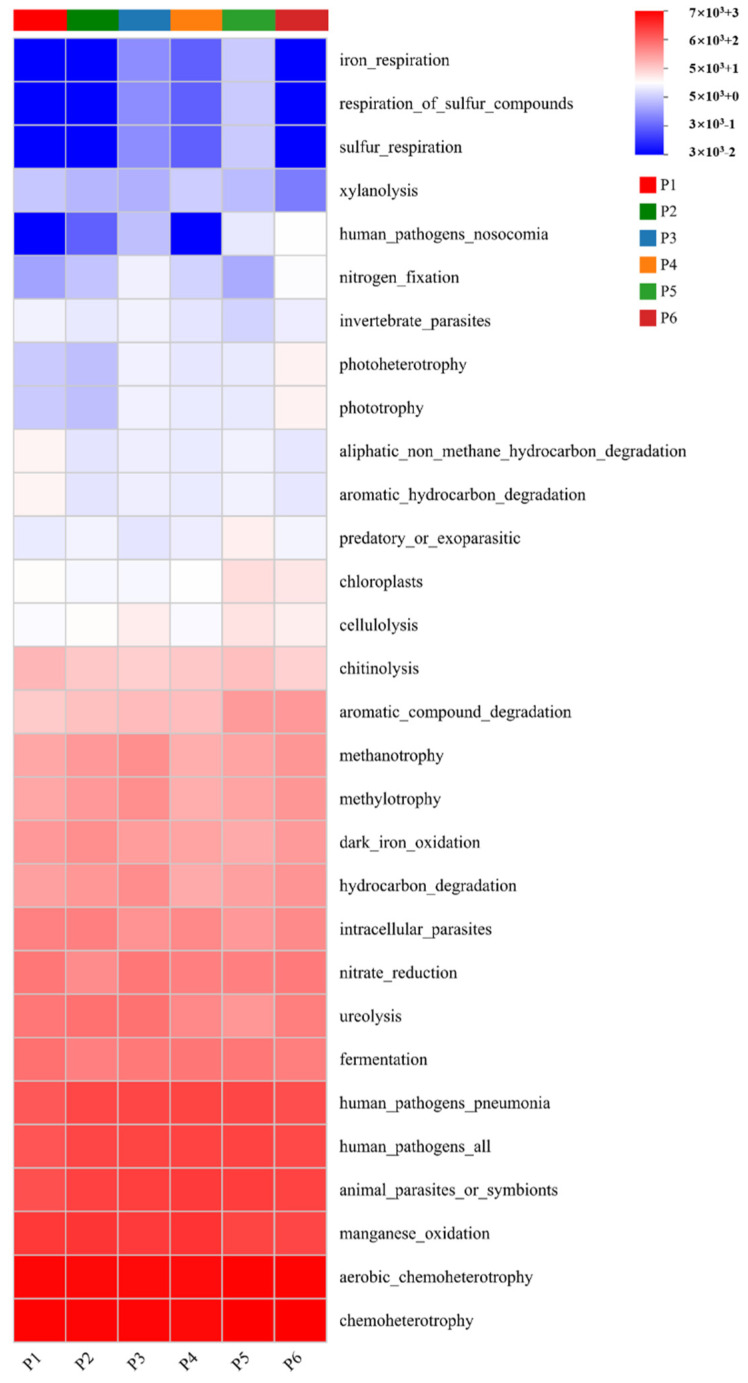
Heatmap of FAPROTAX-predicted functional profiles of soil bacterial communities under different treatments.

Based on 16S rDNA sequencing data, the metabolic functions of bacterial communities under monoculture and mixed-sowing treatments were predicted using FAPROTAX, resulting in the annotation of 30 functional subcategories (Figure 8). The results showed that aerobic chemoheterotrophy and chemoheterotrophy were the dominant metabolic functions among the 30 predicted categories. The functional potential for chemoheterotrophy was highest in treatments P1 (6787), P5 (7125), and P6 (7188), while aerobic chemoheterotrophy was most pronounced in P5 (6652) and P6 (6703). Furthermore, the potential for manganese oxidation was significantly higher in P2 and P3 compared to P1, and the chloroplast functional category was significantly elevated in P5 and P6 relative to P1, likely reflecting greater plant-derived organic inputs in these mixed treatments. Additional analysis revealed significant differences in specific metabolic functions among treatments. The functional potentials for iron respiration, respiration of sulfur compounds, and sulfur respiration were highly expressed in P1, P2, and P6, and significantly higher than in the other treatments. In contrast, the potential for aliphatic non-methane hydrocarbon degradation was significantly higher in P1 and P4 compared to other treatments, suggesting enhanced microbial capacity for degrading certain organic pollutants under these conditions.

### 3.7. Cluster Analysis of Soil Bacterial Communities

**Figure 9:**
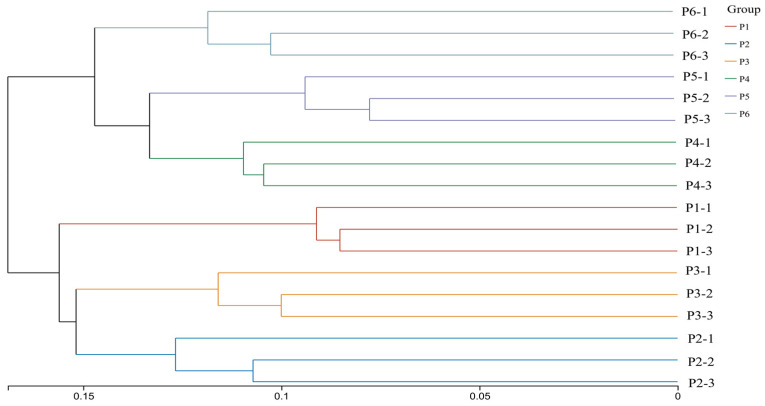
Cluster analysis of soil bacterial communities under different treatments.

Hierarchical clustering analysis was performed based on Bray–Curtis dissimilarity to examine the similarity of soil bacterial communities across the 18 soil samples. As shown in Figure 9, the six treatments could be divided into two distinct groups: Group 1, consisting of treatments P1, P2, and P3; and Group 2, comprising treatments P4, P5, and P6. Further analysis revealed that at a clustering threshold of 0.17, Group 1 could be subdivided into two subgroups: P1 formed a separate subgroup, while P2 and P3 clustered together. At a threshold of 0.15, Group 2 was also divided into two subgroups: P6 formed its own subgroup, whereas P4 and P5 clustered together. Moreover, soil samples from P2 and P3, as well as from P4 and P5, clustered within the same branches, indicating high similarity in bacterial community structure under these mixed-sowing treatments. In contrast, P1 and P6 were located on distant branches, reflecting substantial differences in their bacterial community composition. These results suggest that sowing patterns significantly influence the structure of soil bacterial communities, with certain mixed-species combinations promoting more similar microbial assemblages compared to monoculture or other mixture types.

### 3.8. Coupling Relationships Among Vegetation Community Characteristics, Soil Physicochemical Properties, and Soil Bacterial Communities

**Figure 10:**
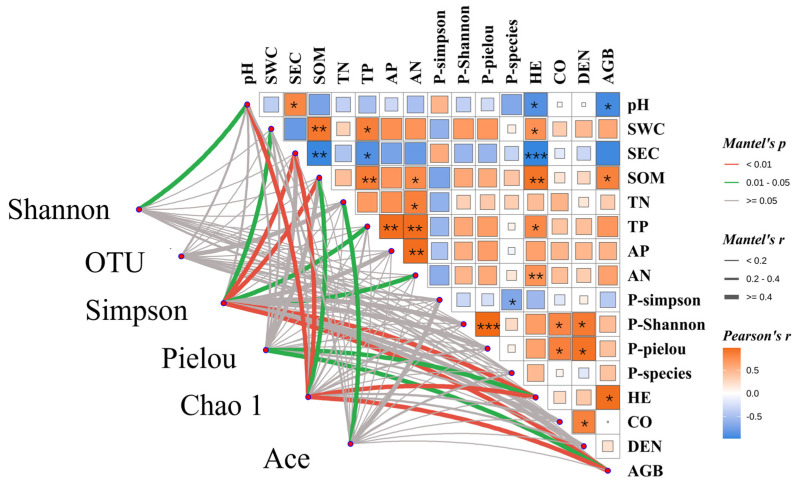
Mantel test analysis of the relationships among vegetation community characteristics, soil physicochemical properties, and soil bacterial community structure. Note: Asterisks denote statistical significance: * *p* < 0.05, ** *p* < 0.01, *** *p* < 0.001. SEC: soil electrical conductivity; SWC: soil water content; SOM: soil organic matter; TN: total nitrogen; TP: total phosphorus; AN: alkali-hydrolyzable nitrogen; AP: available phosphorus; P-Simpson: plant Simpson index; P-Shannon: plant Shannon index; P-Species: plant species richness index; HE: plant height; CO: plant cover; DEN: plant density; AGB: aboveground plant fresh biomass.

A Mantel test was performed to analyze the correlations among soil physicochemical properties, plant community characteristics, and soil bacterial community structure (Figure 10). The results showed that the Shannon index was highly significantly correlated (*p* < 0.01) with SWC, SOM, AP, HE, DEN, and AGB. The number of bacterial OTUs was significantly correlated with TN and CO (*p* < 0.05). The Simpson index showed no significant correlation with the four plant diversity indices (*p* > 0.05). The Pielou evenness index was significantly correlated (*p* < 0.05) with SOM, TN, TP, AP, HE, DEN, and AGB. The Chao1 index showed no significant correlation with TN or the four plant diversity indices (*p* > 0.05), but was significantly (*p* < 0.05) or highly significantly (*p* < 0.01) negatively correlated with other soil physicochemical properties and vegetation characteristics. The Ace index was significantly correlated with SWC and CO (*p* < 0.05).

**Figure 11:**
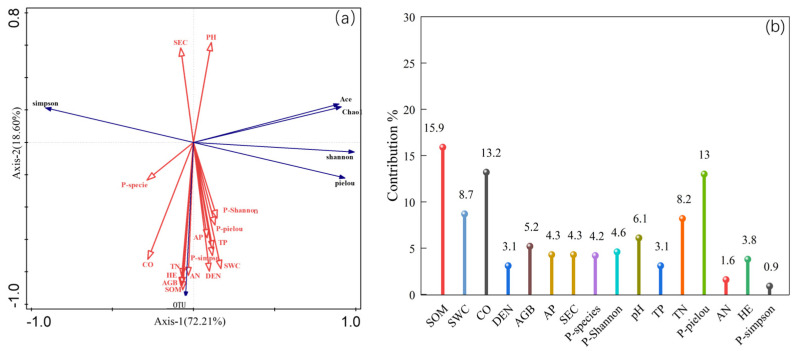
Redundancy analysis (RDA) of the relationships among soil physicochemical properties, plant community characteristics, and soil bacterial community structure. Note: red arrows indicate plant community characteristics and soil factors, and blue arrows indicate the fungal community structure. (**a**) Redundancy Analysis; (**b**) Factor contribution percentage plot. SEC: soil electrical conductivity; SWC: soil water content; SOM: soil organic matter; TN: total nitrogen; TP: total phosphorus; AN: alkali-hydrolyzable nitrogen; AP: available phosphorus; P-Simpson: plant Simpson index; P-Shannon: plant Shannon index; P-Species: plant species richness index; HE: plant height; CO: plant cover; DEN: plant density; AGB: aboveground plant fresh biomass.

Based on redundancy analysis (RDA), the relationships among soil bacterial community diversity, vegetation characteristics, and soil physicochemical factors were analyzed, with the results presented in Figure 11. The explanatory rates of soil bacterial community diversity by plant community characteristics and soil physicochemical properties were 72.21% for axis I and 18.60% for axis II, respectively, with a cumulative explanatory rate reaching 98.81%. SOM, CO, SWC, TN, and PH jointly shaped the community composition. Among these, the arrow representing SOM had the longest vector, with an explanatory rate of 15.8% and a contribution rate of 15.9%, indicating that soil SOM is the most significant soil factor influencing soil bacterial community diversity.

## 4. Discussion

### 4.1. Effects of Monoculture and Mixed Sowing of Graminoids on Vegetation Biomass and Soil Physicochemical Properties in Artificial Grasslands

Establishing artificial grasslands is a common strategy for restoring degraded grasslands. By altering vegetation composition through monoculture or mixed sowing, this approach can improve soil nutrient storage, water retention capacity, and rhizosphere bacterial community structure, thereby influencing fungal community composition and development, ultimately enhancing the ecological functions and sustainable productivity of grasslands [15]. Previous studies have shown that, in degraded grassland restoration, mixed sowing significantly enhances vegetation biomass and soil nutrient levels compared to monoculture [16]. In this study, mixed-sowing treatments exhibited significantly higher vegetation biomass than the monoculture treatment, with the five mixed treatments showing increases ranging from 10.32% to 57.24%. Soil nutrients serve as the fundamental basis for plant growth, and their effective supply directly influences plant development [17]. Our data revealed that mixed sowing significantly increased TN and TP contents, and also exerted positive effects on AN and AP. Moreover, SOM were significantly higher under mixed sowing than monoculture. In particular, the SOM content reached 145.05 g·kg^−1^ in the P4 treatment, indicating that the root architecture and litter input of mixed vegetation enhance soil water retention and aggregate stability, thereby significantly improving soil fertility. Soil SEC and pH were lower in mixed-sowing treatments compared to monoculture. Although the effect on pH was relatively modest, statistically significant differences were observed among treatments, suggesting that root exudates and microbial metabolites may play a role in regulating soil acid-base balance. These findings are consistent with previous studies on mixed sowing in artificial grasslands on the Qinghai–Tibet Plateau, confirming that mixed planting improves nutrient retention and modulates soil pH.

Mixed sowing enhances the soil physicochemical environment in artificial grasslands by increasing vegetation diversity, root biomass, and litter input, thereby promoting the accumulation of soil nutrients, improving water retention, and increasing organic matter content [18]. Additionally, organic acids produced by soil bacterial metabolism not only facilitate the decomposition of organic matter but also neutralize alkaline substances through proton release, creating a microenvironment favorable for nutrient mobilization [19]. Zhang Huimin et al. [20] also confirmed that mixed sowing significantly increases soil nitrogen and organic matter stocks. Li Wen et al. [21] found that a mixture of Elymus nutans, Festuca sinensis, and Poa pratensis not only maintained high productivity but also significantly improved soil nutrient content. Shi Jianjun et al. [22] demonstrated that artificial management practices (fertilization and weed removal) significantly enhanced the productivity of grass mixtures with four or more components, enabling them to maintain a high level of production stability—consistent with the findings of the present study. Notably, the adaptability and stability of mixed grasslands are primarily regulated by sowing ratios, which exhibit significant regional specificity due to variations in local climate and soil conditions [23]. Furthermore, interspecific differences in nutrient uptake efficiency and preferences reinforce this niche differentiation [24]. In summary, mixed sowing alleviates competitive pressure among plant communities for soil nutrients through niche differentiation and heterogeneous nutrient utilization. Therefore, selecting appropriate forage combinations and optimizing sowing ratios are crucial for ensuring the long-term productivity and ecological stability of mixed grasslands [25].

### 4.2. Effects of Monoculture and Mixed Sowing of Graminoids on Soil Bacterial Community Structure and Function in Artificial Grasslands

In both monoculture and mixed-sowing treatments, the dominant bacterial phyla in the soil bacterial community were Pseudomonadota, Acidobacteriota, and Actinomycetota, which exhibited the highest relative abundances, indicating their core functional roles in the soil microbial ecosystem of artificial grasslands. The results demonstrate that heterogeneity in soil nutrient levels significantly shapes bacterial community structure [26]. In this study, Actinomycetota and Acidobacteriota showed a pattern of significant co-enrichment across all treatment groups, maintaining consistently high abundances. This phenomenon is primarily driven by functional complementarity: Actinomycetota are highly efficient in degrading complex organic polymers such as lignin and chitin, thereby playing a key role in carbon cycling, while Acidobacteriota regulate the mineralization and stabilization of organic carbon, contributing to the dynamic balance of soil carbon pools [27]. This synergistic interaction markedly enhances the efficiency of soil carbon transformation and sequestration [28]. This study found that mixed sowing significantly influenced soil bacterial community diversity. In the P4 treatment, compared to the control (P1), the number of OTUs, Shannon index, and Ace index increased significantly by 26.12%, 5.15%, and 25.81%, respectively—consistent with previous studies reporting that mixed sowing enhances bacterial richness and evenness, with average increases in the Shannon index of 5–25% [29,30]. Furthermore, single-factor correlation-based network analysis revealed that the complexity of the rhizosphere bacterial co-occurrence network was significantly enhanced in the five mixed-sowing treatments compared to monoculture, with a higher proportion of positive correlations than in the P1 treatment. This suggests that in mixed-sowing systems, most bacterial taxa form tightly coordinated networks through mutualistic interactions and cooperative coexistence. Such highly connected microbial structures can optimize nutrient turnover efficiency in the rhizosphere and accelerate the synchronized metabolic activities of nitrogen-fixing and phosphorus-solubilizing bacteria [31].

Alterations in soil bacterial community structure can lead to significant differences in functional characteristics of soil bacterial communities [32]. In this study, bacterial functional profiles under different sowing treatments were predicted using FAPROTAX, revealing distinct functional patterns among the six treatments—consistent with previous findings. Notably, the potential for manganese oxidation was significantly higher in treatments P2 and P3 compared to others (*p* < 0.05). The underlying mechanism is as follows: mixed-sowing systems enhance complementary resource use and optimize the rhizosphere microenvironment through interspecific plant interactions, thereby improving overall photosynthetic efficiency [33]. Additionally, the functional potential associated with chloroplasts—a proxy for photosynthetic activity—was significantly elevated in mixed-sowing treatments, further supporting the above hypothesis. In contrast, the monoculture treatment (P1) exhibited lower functional potential related to photoautotrophy, indicating that, compared to mixed sowing, monoculture systems suffer from limited organic matter input, resulting in severe shortages of substrates (e.g., sulfides, low-molecular-weight organics) required for the growth and metabolic activity of phototrophic bacteria.

### 4.3. Response Associations Among Rhizosphere Microbial Composition, Soil Physicochemical Properties, and Plant Community Characteristics

Mantel test analysis revealed an extremely significant positive correlation (*p* < 0.01) between soil pH and soil electrical conductivity (SEC), and both variables showed extremely significant negative correlations (*p* < 0.01) with other soil physicochemical properties and plant community characteristics, including vegetation CO, HE, DEN, and AGB. This finding is consistent with previous studies, indicating that higher soil nutrient and moisture levels promote better plant community growth and development. Redundancy analysis (RDA) showed that soil SEC was negatively correlated with bacterial diversity indices—specifically the Shannon index, Pielou evenness index, and OTU richness—suggesting that soil salinity stress significantly suppresses the recovery and maintenance of bacterial community diversity [34]. In contrast, most plant community characteristics and soil fertility indicators—such as SWC, TN, and TP—were significantly positively correlated with bacterial diversity indices. This indicates that organic matter and mineral nutrients (N and P) can promote bacterial community succession and functional differentiation by providing energy sources and metabolic substrates, thereby facilitating the restoration of microbial diversity [35]. In summary, during the restoration of alpine artificial grasslands, a synergistic “plant–soil–microbe” interaction mechanism enables bidirectional enhancement between soil fertility improvement and bacterial community optimization, ultimately establishing a self-reinforcing, positive ecological feedback loop.

Soil physicochemical properties exert a significant shaping effect on the diversity and structure of soil bacterial communities in alpine artificial grasslands in Qinghai Province. Contrary to traditional views that emphasize soil nutrient availability as the primary driver of bacterial community distribution, this study highlights the particularly prominent role of SOM. Based on multiple lines of evidence—including 16S rRNA gene sequencing and RDA analysis—SOM emerged as the most significant and influential factor affecting bacterial diversity. This finding aligns with previous studies, in which SOM typically explains 20–40% of bacterial community variation—more than other factors such as pH or TN—because it directly supplies carbon substrates and shapes microhabitat heterogeneity, thereby promoting bacterial niche differentiation. Under alpine conditions, SOM enhances soil moisture retention and thermal stability, mitigating the inhibitory effects of freeze–thaw cycles on microbial activity and helping to maintain bacterial community stability [36,37]. Overall, this process establishes a positive feedback loop: higher bacterial diversity accelerates SOM mineralization, thereby maximizing microbially driven ecological functions such as carbon cycling and nutrient transformation, which in turn support sustainable grassland restoration.

## 5. Conclusions and Prospects

This study demonstrates that mixed sowing, particularly the four-species combination (P4 treatment) of Elymus nutans, Poa pratensis, Poa crymophila, and Festuca sinensis, can synergistically enhance ecosystem functions in the ecological restoration of degraded alpine grasslands through a “vegetation-soil-microorganism” interaction mechanism. The P4 treatment not only significantly increased aboveground biomass and soil organic matter (SOM) content but also optimized the rhizosphere bacterial community structure, enhanced bacterial diversity, and promoted beneficial phyla such as Actinomycetota and Acidobacteriota to become dominant functional groups. Soil organic matter was identified as a key environmental factor driving bacterial community assembly.

Looking forward, these findings support the promotion of multi-species mixed sowing, especially the P4 combination, as an effective strategy for sustainable grassland restoration in degraded alpine regions. Future research should focus on optimizing species mixing ratios and elucidating the specific roles and regulatory pathways of key beneficial bacterial phyla (e.g., Actinomycetota and Acidobacteriota) in critical ecological processes such as carbon and nitrogen cycling. This will provide a theoretical foundation for enhancing the service functions of artificial grassland ecosystems.

## Figures and Tables

**Table 1:** Configuration of mixed-sowing treatments.

Treatment	Grass Species Combination	Mixed Sowing Ratio
P1	*Elymus nutans*	1
P2	*Elymus nutans* + *Poa pratensis*	1:1
P3	*Elymus nutans* + *Poa pratensis* + *Festuca sinensis*	1:1:1
P4	*Elymus nutans* + *Poa pratensis* + *Festuca sinensis*+ *Poa crymophila*	1:1:1:1
P5	*Elymus nutans* + *Poa pratensis* + *Festuca sinensis*+ *Poa crymophila* + *Puccinellia distans*	1:1:1:1:1
P6	*Elymus nutans* + *Poa pratensis* + *Festuca sinensis*+ *Poa crymophila* + *Puccinellia distans*+ *Festuca kryloviana* cv. Huanhu	1:1:1:1:1:1

**Table 2:** Changes in vegetation community characteristics under different treatments.

Treatment	Simpson Diversity Index	Shannon-Wiener Diversity Index	Pielou Evenness Index	Species Richness Index	Plant Heigh t/cm	Plant Coverage /%	Densit y/Plant·m^−2^	Aboveground Biomass /(g·m^−2^)
P1	0.88 ± 0.02 a	1.96 ± 0.06 b	0.67 ± 0.00 b	2.33 ± 0.15 b	17.60 ± 1.19 e	76.30 ± 2.28 c	599.33 ± 19.67 c	239.00 ± 9.68 c
P2	0.86 ± 0.00 a	2.25 ± 0.06 a	0.75 ± 0.00 a	2.48 ± 0.12 a	22.97 ± 1.49 d	81.13 ± 1.39 b	806.67 ± 14.53 bc	241.60 ± 4.10 b
P3	0.84 ± 0.00 ab	2.26 ± 0.05 a	0.76 ± 0.01 a	2.59 ± 0.12 a	25.37 ± 0.69 cd	83.33 ± 0.52 b	1060.00 ± 47.30 ab	270.90 ± 9.37 b
P4	0.82 ± 0.00 b	2.19 ± 0.02 a	0.74 ± 0.00 a	2.45 ± 0.14 a	32.53 ± 1.29 a	88.33 ± 0.84 a	1223.37 ± 86.85 a	375.80 ± 14.73 a
P5	0.88 ± 0.00 a	2.28 ± 0.05 a	0.77 ± 0.02 a	2.53 ± 0.04 a	29.70 ± 0.97 ab	82.97 ± 0.96 b	1233.03 ± 45.29 a	371.27 ± 45.58 a
P6	0.86 ± 0.01 a	2.20 ± 0.04 a	0.76 ± 0.00 a	2.30 ± 0.07 b	28.23 ± 1.41 bc	81.70 ± 1.45 b	1106.67. ± 58.12 ab	342.93 ± 6.16 ab

Note: Data are presented as mean ± standard deviation; different lowercase letters within the same column indicate significant differences between treatments at the *p* < 0.05 level.

**Table 3:** Changes in soil physicochemical properties under different treatments.

Treatment	SWC/%	SEC/(μs·cm^−1^)	pH	SOM/(g·kg^−1^)	TN/(g·kg^−1^)	TP/(g·kg^−1^)	AN/(mg·kg^−1^)	AP/(mg·kg^−1^)
P1	18.11 ± 1.36 c	495.83 ± 28.41 a	7.55 ± 0.16 a	86.21 ± 15.08 e	3.98 ± 0.09 d	1.47 ± 0.21 d	43.86 ± 0.19 d	34.15 ± 2.33 c
P2	21.30 ± 0.77 b	458.50 ± 7.27 b	7.52 ± 0.05 a	108.42 ± 10.09 d	4.05 ± 0.25 d	1.57 ± 0.15 d	49.17 ± 3.42 c	35.60 ± 1.65 c
P3	22.01 ± 0.31 ab	411.13 ± 44.99 c	7.44 ± 0.18 a	128.67 ± 4.01 c	4.35 ± 0.18 c	1.92 ± 0.12 c	50.63 ± 1.49 c	37.77 ± 1.52 c
P4	23.77 ± 1.71 a	375.70 ± 32.49 d	7.02 ± 0.22 a	145.05 ± 5.37 a	6.92 ± 0.12 a	2.29 ± 0.06 a	61.50 ± 1.73 a	41.30 ± 1.71 ab
P5	24.58 ± 0.29 a	436.63 ± 6.59 bc	7.18 ± 0.15 a	135.99 ± 7.38 ab	6.68 ± 0.44 ab	2.01 ± 0.10 b	56.50 ± 2.93 b	40.93 ± 1.51 ab
P6	25.44 ± 1.71 a	393.13 ± 34.97 c	7.41 ± 0.10 a	147.43 ± 12.30 a	5.50 ± 0.18 b	2.40 ± 0.05 a	60.37 ± 2.15 ab	43.57 ± 0.84 a

Note: Data are presented as mean ± standard deviation; different lowercase letters within the same column indicate significant differences between treatments at the *p* < 0.05 level.

## Data Availability

The original contributions presented in this study are included in the article. Further inquiries can be directed to the corresponding author.
